# Refractory livedoid vasculopathy successfully treated with immunosuppressive therapy

**DOI:** 10.1016/j.jdcr.2025.10.012

**Published:** 2025-10-14

**Authors:** Andres Chaponan-Lavalle, Cherie Quiroz Cortegana, Jose M. Estrada-Grossmann, Elar Quispe Mena, Nelson Diaz, Guichell Revilla Robinson

**Affiliations:** a Departamento de Medicina, Universidad Peruana de Ciencias Aplicadas, Lima, Perú; b Departamento de Medicina Interna, Clinica Good Hope, Lima, Perú; c Departamento de Medicina, Universidad Peruana Unión, Lima, Perú

**Keywords:** immunosuppressive, livedoid vasculopathy, thrombotic vasculopathy, ulcer

## Introduction

Livedoid vasculopathy (LV) is a rare thrombo-occlusive condition with an estimated prevalence of 1 in 100, 000 per year. It most commonly affects individuals between the ages of 15-50 years, with a female to male ratio of 3:1. The condition was first described by Feldaker et al in 1955 as “livedo reticularis with summer ulcerations.” However, LV is now the preferred term, as it more accurately reflects the disease's thrombotic nature without evidence of true vasculitis.

LV is characterized by painful ulcer, atrophie blanche, and livedo racemose, primarily affecting the lower limbs.^1^ Diagnostic delays, median of 3.4 years, are common due to nonspecific clinical signs and limited familiarity among healthcare providers.^2^ While anticoagulation is standard in thrombotic forms, some patients fail to respond and may require alternative approaches.^3^

We report a case of refractory LV successfully managed with low dose corticosteroids and hydroxychloroquine, supporting the role of immunosuppressive therapy in selected cases.

## Case history

**Fig 1:**
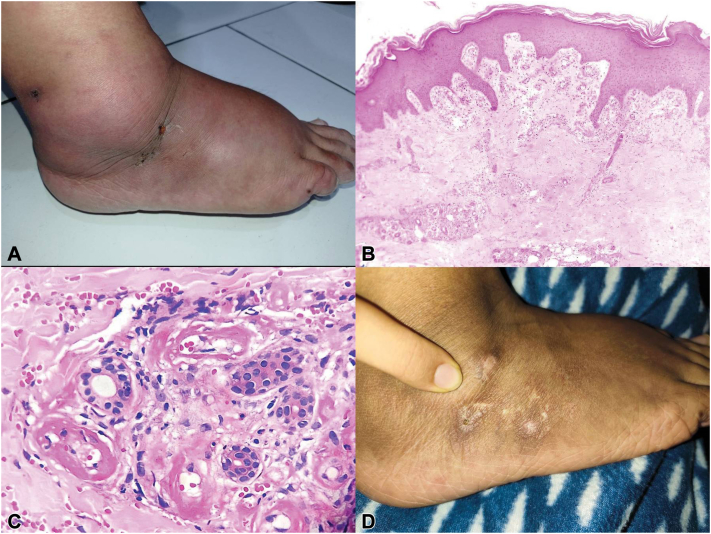
**A,** Initial clinical presentation with ulcers of irregular borders on the medial malleolus and surrounding edema, accompanied by purpuric macules. **B,** Histopathology showing neovascularization, telangiectasias, and occluded superficial and mid dermal vessels without signs of vasculitis. **C,** High power view of hyalinized dermal vessels with fibrin deposition and red blood cells extravasation, without evidence of inflammatory cells or karyorrhexis. **D,** Post-treatment resolution of lesions following low dose corticosteroids and hydroxychloroquine.

A 37-year-old woman presented with symmetric progressive burning sensation and weakness on lower extremities for the last 6 months. She was initially treated with antibiotics for presumed cellulitis without improvement. Physical examination showed irregular ulcers in the medial malleolar regions of the right foot, with bilateral nonpitting edema and reticular purpuric macules (Fig 1, *A*). No upper extremities involvement was noted.

Laboratory finding included thrombocytosis (478 × 10^3^/μL), normal inflammatory markers (CRP 3.75 mg/L, ESR 15 mm/h) and a prolonged aPTT (60.3 sec) with normal antithrombin III levels and a negative lupus anticoagulant test. Infectious (HBsAg, HIV, VDRL) and autoimmune serologies (ANA, ANCA panel, and antiphospholipid antibodies) were unremarkable. Platelet aggregation studies were within normal limits. Skin biopsy revealed fibrin thrombi in superficial and deep dermal vessels, dermal fibrosis and red blood cell extravasation, without inflammatory infiltrate or karyorrhexis (Fig 1, *B* and *C*).

The patient was initially treated with Rivaroxaban (20 mg/d) but was discontinued because of lack of response. The patient was started on prednisone (5 mg twice daily) with partial clinical improvement. Hydroxychloroquine (200 mg/d) was added, leading to resolution of ulcers, edema, and significant pain relief (Fig 1, *D*).

## Discussion

LV is a rare, chronic thrombo-occlusive disorder primarily affecting the lower extremities. The clinical presentation is often bilateral, characterized by livedoid changes (erythema to purple macules, papules, patches or nodules) that evolve into atrophie blanche (white colored atrophic plaques surrounded by hyperpigmentation and telangiectasias) and often accompanied by intensely painful ulcerations.^1^ LV typically follows a chronic, relapsing remitting course, with flares lasting 55 days and recurring every 8 months.^2^^,^^3^

LV is believed to result from microvascular thrombosis, endothelial injury, and impaired fibrinolysis.^4^ Diagnosing livedoid vasculopathy requires histopathologic confirmation taken from the edge of a fresh ulcer or a purpuric lesion. Histopathologic findings include intraluminal thrombosis, endothelial proliferation, and subintimal hyaline degeneration. Direct immunofluorescence studies often reveal deposits of C3 and immunoglobulin M within dermal blood vessels and at the dermal-epidermal junction.^4^^,^^5^

**Fig 2:**
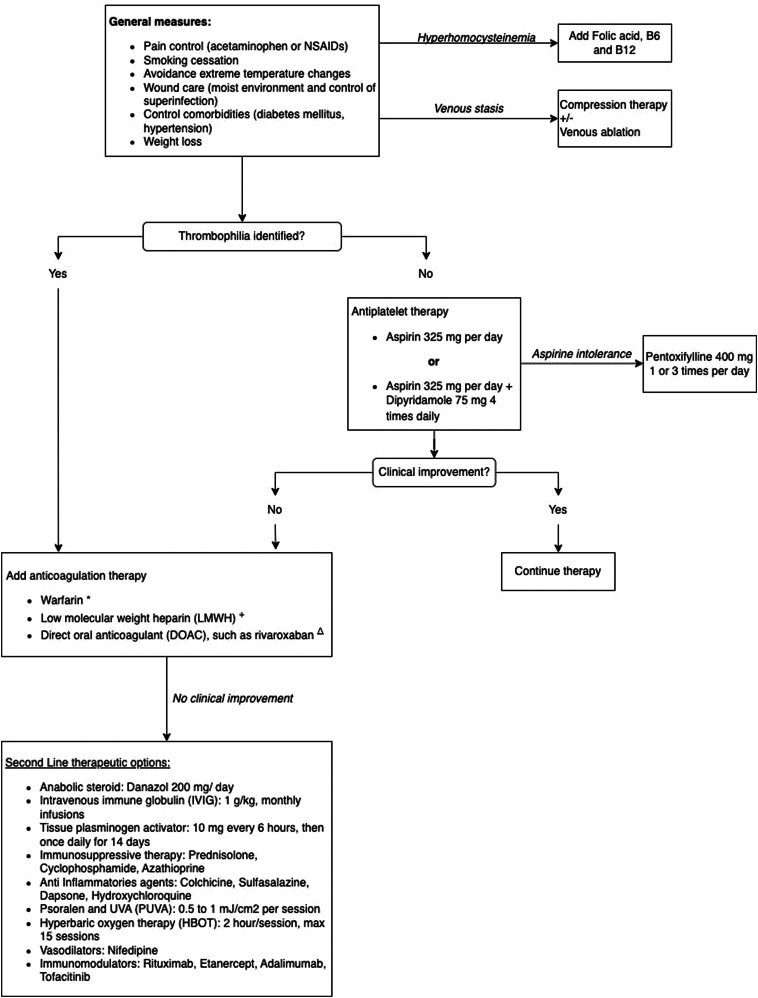
Proposed treatment algorithm for livedoid vasculopathy: ∗Warfarin is adjusted to maintain an INR between 2 and 3; clinical improvement is often observed within 2 months. ^+^Enoxaparin is administered subcutaneously at 1 mg/kg every 12 hours for 6 months, then once daily. ^Δ^Rivaroxaban is administered at 10 mg twice daily, with the option to reduce to once daily upon clinical improvement. *INR*, International normalization ratio.

Therapeutic approaches (Fig 2) focus on adequate pain control and wound care emphasizes maintaining a moist environment to promote healing and prevent superinfection. The first-line therapy for patients without an identified thrombophilia is an antiplatelet agent, such as aspirin alone for initial therapy or in combination with dipyridamole. Pentoxifylline can be used in patients who cannot tolerate aspirin. While anticoagulants remain the cornerstone in thrombotic presentations, their efficacy varies, particularly in idiopathic or autoimmune-associated forms. Warfarin is adjusted to maintain an international normalization ratio between 2 and 3 and may lead to marked clinical improvement within the first 2 months of therapy. LMWH is administered subcutaneously at 1 mg/kg every 12 hours for 6 months followed by once daily dosing. Rivaroxaban (10-20 mg/d) has demonstrated 82.2% efficacy in LV, both in patients with or without thrombophilia, achieving pain and ulcer remission.^6^

Recent literature supports the role of immunomodulatory and alternative therapies in refractory LV. Among pharmacological agents, anabolic steroids and intravenous immunoglobulin have demonstrated efficacy, particularly in LV associated with connective tissue disease.^7^ Danazol, the second most frequently prescribed agent, enhances fibrinolysis and protein C/S synthesis, though its use is restricted to short courses (4-12 weeks) due to androgenic and metabolic adverse effects. Intravenous immunoglobulin, administered as monthly infusions (1-2 g/kg), has achieved remission in up to 95% of refractory and newly diagnosed cases. Tissue plasminogen activator may be considered in patients resistant to anticoagulants, given as a short IV regimen followed by maintenance antiplatelet or anticoagulant therapy. Immunosuppressive agents, including corticosteroids, cyclophosphamide, and azathioprine, can be added to conventional regimens to enhance efficacy, while anti-inflammatory drugs such as colchicine, dapsone, sulfasalazine, doxycycline, and hydroxychloroquine serve as useful adjuvants.^6^ In patients with hyperhomocysteinemia, supplementation with folate, pyridoxine, and hydroxocobalamin is recommended. Local and adjunctive therapies include hyperbaric oxygen therapy and PUVA, both of which improve tissue oxygenation and angiogenesis, although their use is limited by accessibility. In patients with concomitant venous insufficiency, compression or venous ablation may facilitate ulcer healing by lowering venous hypertension.^5^ Collectively, these therapeutic options support the need for an individualized approach in refractory LV.

Our patient’s lack of response to anticoagulation and subsequent improvement with low-dose immunosuppression suggests a possible autoimmune or inflammatory component in her disease. Although skin biopsy did not reveal frank vasculitis, the clinical pattern and therapeutic response align with reports advocating individualized treatment strategies. This case contributes to growing evidence that anti-inflammatory therapy may benefit patients with LV resistant to anticoagulation alone.

## Conflicts of interest

None disclosed.

## References

[1] Seguí M., Llamas-Velasco M. (2022). A comprehensive review on pathogenesis, associations, clinical findings, and treatment of livedoid vasculopathy. Front Med (Lausanne).

[2] Gardette E., Moguelet P., Bouaziz J. (2018). Livedoid vasculopathy: a French observational study including therapeutic options. Acta Derm Venerol.

[3] Gao Y., Jin H. (2021). Rivaroxaban for treatment of livedoid vasculopathy: a systematic review. Dermatol Ther.

[4] Burg M.R., Mitschang C., Goerge T. (2022). Livedoid vasculopathy - a diagnostic and therapeutic challenge. Front Med (Lausanne).

[5] Bilgic A., Ozcobanoglu S., Bozca B.C. (2021). Livedoid vasculopathy: a multidisciplinary clinical approach to diagnosis and management. Int J Womens Dermatol.

[6] Micieli R., Alavi A. (2018). Treatment for livedoid vasculopathy: a systematic review. JAMA Dermatol.

[7] Eswaran H., Googe P., Vedak P. (2022). Livedoid vasculopathy: a review with focus on terminology and pathogenesis. Vasc Med.

